# Supplementary material for: Aspirin Is Related to Worse Clinical Outcomes of COVID-19
Source: Medicina (Kaunas). 2021 Sep 4;57(9):931. doi: 10.3390/medicina57090931 (PMC8465059; doi:10.3390/medicina57090931)
Supplement: Supplementary file 1 [file medicina-57-00931-s001.zip › medicina-1326758-supplementary.pdf]

Table S1. Coding for underlying disease.

Diseases	ICD-10 Codes
Hypertension	I10, I11, I12, I13, I15 *
Chronic obstructive pulmonary diseases	J44, J43 (except J43.0) †
Asthma	J45, J46 ‡
Chronic kidney disease	N03, N05, N165, N18, N19, N250, I12, I13, Z490, Z491, Z492, Z940, Z992, E102, E112, E132, E142, T861 ‖
Diabetes mellitus	E10, E11, E12, E13, E14 *
Cerebrovascular disease	G45, G46, I60, I61, I62, I63, I64, I65, I66, I67, I68, I69, H340 *

ICD, International Classification of Disease;

* Jung, S. Y. , Choi, J. C., You, S. H., Kim, W. Y. Association of Renin-angiotensin-aldosterone System Inhibitors With Coronavirus Disease 2019 (COVID-19)- Related Outcomes in Korea: A Nationwide Population-based Cohort Study. Clinical Infectious Diseases 2020;71(16):2121–8. doi: 10.1093/cid/ciaa624

† Lee, S.C., Son, K. J., Han, C. H., Park S. C., Jung, J. Y. Impact of COPD on COVID-19 prognosis: A nationwide population-based study in South Korea. Scientific Reports 11, 3735 (2021). doi.org/10.1038/s41598-021-83226-9

‡ Woo, A., Lee, S. W., Koh, H. Y., Kim, M. A., Han, M. Y., & Yon, D. K. (2020). Incidence of cancer after asthma development: two independent population-based cohort studies. Journal of Allergy and Clinical Immunology. doi:10.1016/j.jaci.2020.04.041

‖ Kang, S. H., Kim, S. W., Kim, Y., Cho, K. H., Park, J. W., Do, J. Y. Association between Chronic Kidney Disease or Acute Kidney Injury and Clinical Outcomes in COVID-19 Patients. J Korean Med Sci. 28;35(50). doi.org/10.3346/jkms.2020.35.e434
